# Molecular Drivers of Onco*type* DX, Prosigna, EndoPredict, and the Breast Cancer Index: A TransATAC Study

**DOI:** 10.1200/JCO.20.00853

**Published:** 2020-10-27

**Authors:** Richard Buus, Ivana Sestak, Ralf Kronenwett, Sean Ferree, Catherine A. Schnabel, Frederick L. Baehner, Elizabeth A. Mallon, Jack Cuzick, Mitch Dowsett

**Affiliations:** ^1^ The Breast Cancer Now Toby Robins Research Centre at The Institute of Cancer Research, London, United Kingdom; ^2^ Ralph Lauren Centre for Breast Cancer Research, Royal Marsden Hospital, London, United Kingdom; ^3^ Centre for Cancer Prevention, Wolfson Institute of Preventive Medicine, Queen Mary University of London, London, United Kingdom; ^4^ Myriad International GmbH, Cologne, Germany; ^5^ NanoString Technologies, Seattle, WA; ^6^ Biotheranostics, San Diego, CA; ^7^ Genomic Health, Redwood City, CA; ^8^ Department of Pathology, University of California, San Francisco, San Francisco, CA; ^9^ Department of Pathology, Southern General Hospital, Glasgow, United Kingdom

## Abstract

The Onco*type* DX Recurrence Score (RS), Prosigna Prediction Analysis of Microarray 50 (PAM50) Risk of Recurrence (ROR), EndoPredict (EP), and Breast Cancer Index (BCI) are used clinically for estimating risk of distant recurrence for patients receiving endocrine therapy. Discordances in estimates occur between them. We aimed to identify the molecular features that drive the tests and lead to these differences.

**PATIENTS AND METHODS:**

Analyses for RS, ROR, EP, and BCI were conducted by the manufacturers in the TransATAC sample collection that consisted of the tamoxifen or anastrozole arms of the ATAC trial. Estrogen receptor–positive/human epidermal growth factor receptor 2 (HER2)–negative cases without chemotherapy treatment were included in which all four tests were available (n = 785). Clinicopathologic features included in some tests were excluded from the comparisons. Estrogen, proliferation, invasion, and HER2 module scores from RS were used to characterize the respective molecular features. Spearman correlation and analysis of variance tests were applied.

**RESULTS:**

There were moderate to strong correlations among the four molecular scores (ρ = 0.63-0.74) except for RS versus ROR (ρ = 0.32) and RS versus BCI (ρ = 0.35). RS had strong negative correlation with its estrogen module (ρ = −0.79) and moderate positive correlation with its proliferation module (ρ = 0.36). RS’s proliferation module explained 72.5% of ROR’s variance, while the estrogen module explained only 0.6%. Most of EP’s and BCI’s variation was accounted for by the proliferation module (50.0% and 54.3%, respectively) and much less by the estrogen module (20.2% and 2.7%, respectively).

**CONCLUSION:**

In contrast to common understanding, RSs are determined more strongly by estrogen-related features and only weakly by proliferation markers. However, the EP, BCI, and particularly ROR scores are determined largely by proliferative features. These relationships help to explain the differences in the prognostic performance of the tests.

## INTRODUCTION

During the past 15 years, several multiparameter genomic tests have entered mainstream care for patients with early breast cancer, with some being endorsed for use by authoritative guidelines groups (ASCO, National Institute for Health and Care Excellence).^1,2^ The predominant use of the tests is in the management of estrogen receptor (ER)–positive primary disease. All approved tests show prognostic ability that is beyond that provided by standard clinicopathologic factors such that patients in whom the tests indicate excellent prognosis may safely be excluded from the administration of chemotherapy. The Onco*type* DX Recurrence Score (RS; Genomic Health, Redwood City, CA) has 16 genes that characterize tumor biology along with five reference genes and has been the most widely used test.

**CONTEXT**

**Key Objective**
We investigated the reasons for the frequently observed differences in estimates of risk of distant recurrence provided by four widely used commercial molecular profiling tests—Onco*type* DX Recurrence Score (RS), Prosigna Risk of Recurrence (ROR), EndoPredict (EP), and Breast Cancer Index (BCI)—in the TransATAC cohort.
**Knowledge Generated**
There were strong correlations among the four scores except for RS versus ROR and RS versus BCI. The differences among the tests were due to RS, against expectations, being dominated by its information on estrogen signaling, and ROR, also against expectations, being dominated by information on proliferation, while there was an intermediate position for the EP and BCI.
**Relevance**
These results should help clinicians to understand the reasons for different estimates of risk for individual patients and may allow them to give greater weighting to one result over another, depending on the clinical context.

Other tests include the Prediction Analysis of Microarray 50 (PAM50) Risk of Recurrence (ROR) score (Prosigna; NanoString Technologies, Seattle, WA),^3^ EndoPredict (EP; Myriad Genetics, Cologne, Germany),^4^ Breast Cancer Index (BCI; Biotheranostics, San Diego, CA),^5^ and MammaPrint^6^ (Agendia, Amsterdam, the Netherlands) that measure 46, 8, 7, and 70 genes, respectively, to characterize tumor biology in addition to reference genes. All but the last of these provide an estimate of residual risk of distant recurrence on the basis that patients will receive 5 years of adjuvant endocrine therapy. MammaPrint provides a prognostic estimate if no adjuvant treatment is to be administered. Understanding the molecular drivers of each of these tests and how they differ among the tests is key to interpreting discrepant estimates of risk that are made in many cases.^7,8^

It is widely believed that the RS is dominated by its proliferation module because five of the prognostic 16 genes in the test sit in the proliferation module and because it has the largest coefficient of each of the components of the integrative algorithm.^9^ An analysis of publicly available gene expression data from multiple studies involving approximately 3,000 patients supported the view of the importance of proliferation genes in the RS.^10^ It should be noted, however, that a threshold is applied to the numeric value given to the prognostic module such that only those cases with a score above that threshold are differentiated from one another on the basis of proliferation.^9^ We have drawn upon the TransATAC data set of 785 ER-positive/human epidermal growth factor receptor 2 (HER2)–negative primary breast cancers to examine the degree to which proliferation does in fact drive the RS and other molecular tests in their commercial form as well as consider other features of the tests that could explain discordances.

## PATIENTS AND METHODS

Data were available from TransATAC, a translational study of samples collected from patients with hormone receptor–positive early-stage breast cancer treated with 5 years of tamoxifen or anastrozole in the ATAC randomized clinical trial.^11^ Women were excluded from this analysis if they received chemotherapy or had HER2-positive disease. This study was approved by the South East London Research Ethics Committee. All patients provided written informed consent for their tissue to be used in translational research.

RNA was extracted as described before.^12^ The four tests included in the current study were the RS, EP, Prosigna ROR, and BCI. Of note, the last three tests include clinical factors in their overall score (EP and ROR for all patients, BCI for node-positive disease), but for the purposes of this study, only the molecular component of the respective score was assessed. Molecular analyses were conducted by the commercial providers of the respective scores using RNA extracted by Genomic Health.^12^ The relationship of the risk estimates with prognosis in the TransATAC sample set has been described before.^12-15^ For a sample to be included in the current analysis, data on all four prognostic scores had to be available.

To study associations between continuous variables, Spearman’s rank correlation was used. An analysis of variance of components of the RS score was conducted. Genes that constitute the RS modules are listed in Appendix Table A1 (online only). All statistical analyses were performed with R 3.6.1 software (R Foundation for Statistical Computing, Vienna, Austria).

## RESULTS

Molecular and clinical data were available for analysis from 785 samples. Clinical characteristics of this cohort are listed in Table 1. All patients were postmenopausal at diagnosis; 55.8% of the tumors were < 2 cm, and 21.4%, 60.3%, and 18.3% were low, intermediate, and high grade, respectively. The distribution of the molecular scores and the modules of the RS are listed in Appendix Table A2 (online only). The median values were 15.3 (interquartile range [IQR], 10.2-22.7) for RS, 40.2 (IQR, 23.5-56.3) for ROR, 5.5 (IQR, 4.2-7.0) for EP, and 4.8 (IQR, 3.7-5.9) for BCI. Using the original cutoffs, RS categorized 481, 222, and 82 patients into the low-, intermediate-, and high-risk groups, respectively. On the basis of the cutoffs used in the TAILORx trial, there were 231, 412, and 142 patients in the low-, intermediate-, and high-risk categories, respectively.

**TABLE 1.**
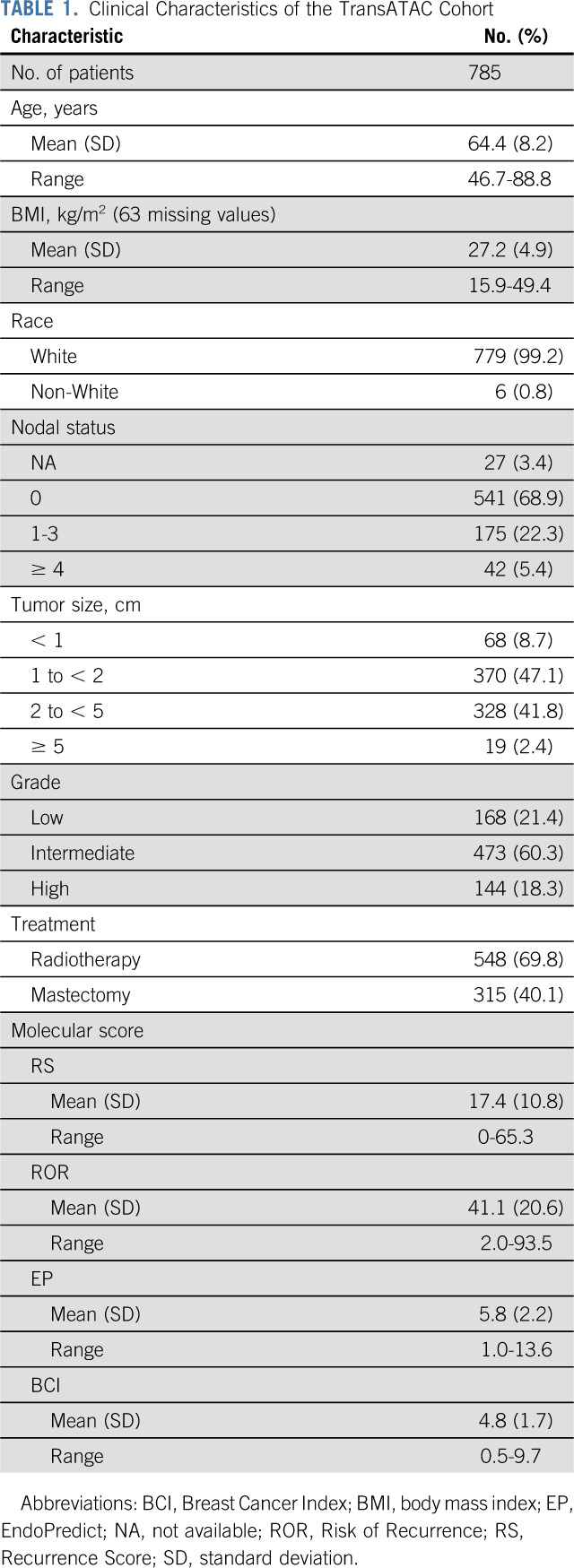
Clinical Characteristics of the TransATAC Cohort

### Relationship of RS, ROR, EP, and BCI Molecular Scores With Clinicopathologic Characteristics

**FIG 1.**
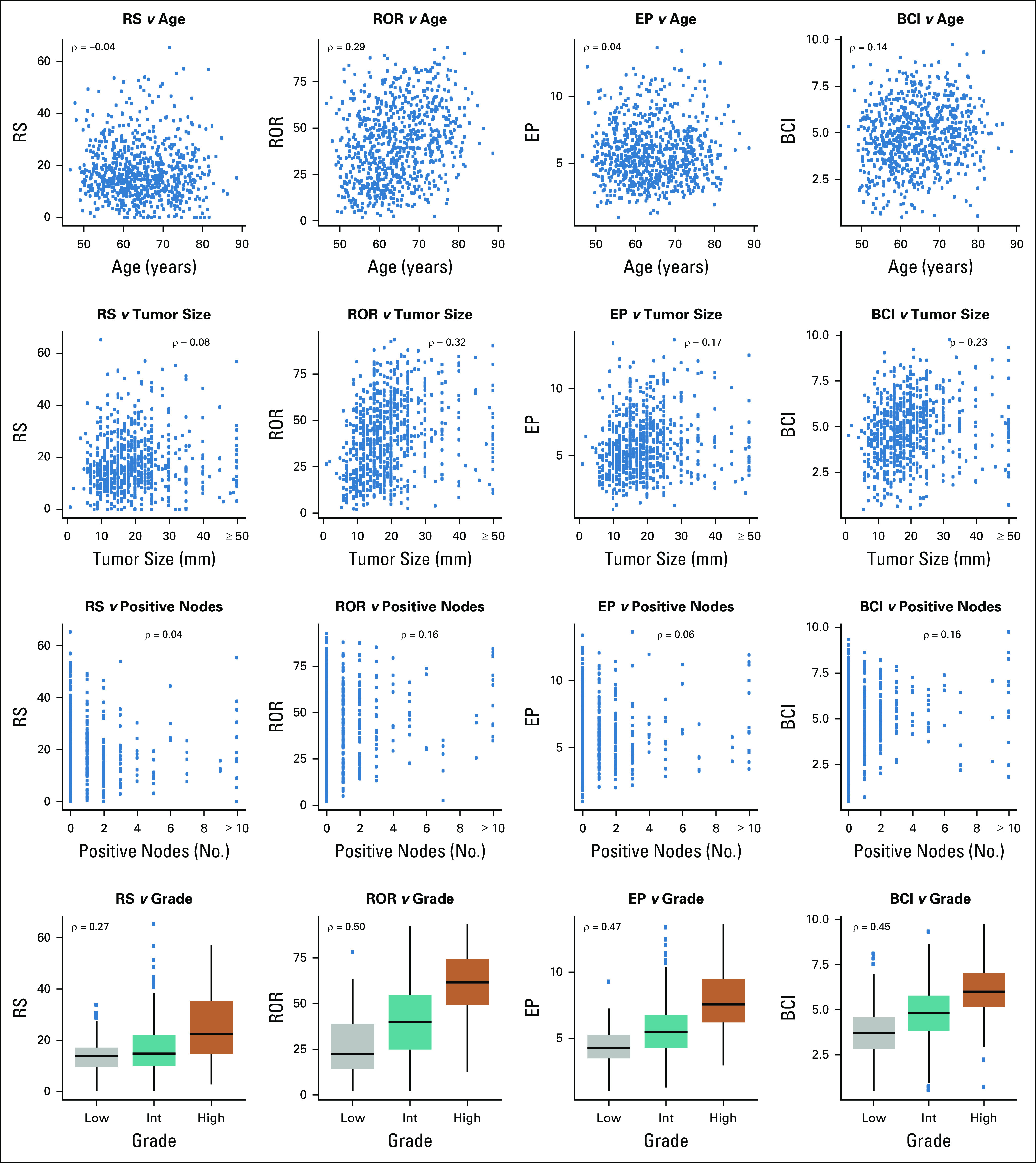
Relationship of the Recurrence Score (RS), Risk of Recurrence (ROR), EndoPredict (EP), and Breast Cancer Index (BCI) molecular scores with clinical characteristics. Number of positive nodes > 10 was set to 10 in this figure. Spearman’s ρ correlation coefficients are presented. Tumor sizes > 50 mm were set to 50 in this figure. Int, intermediate.

The correlation of each of the scores with patient age, tumor size, nodal status, and grade is shown in Figure 1. Each of the scores showed a statistically significant relationship with grade. This was weak for the RS (ρ = 0.27) but moderate and similar for ROR, EP, and BCI (ρ = 0.45-0.50). RS and EP showed no correlation > 0.20 with age, tumor size, or nodal status. However, the ROR correlated weakly and moderately with age and tumor size (ρ = 0.29 and 0.32, respectively), and the BCI had a correlation of 0.23 with tumor size. None of the signatures showed substantive correlations with nodal status.

### Relationship Among RS, ROR, EP, and BCI Molecular Scores

**FIG 2.**
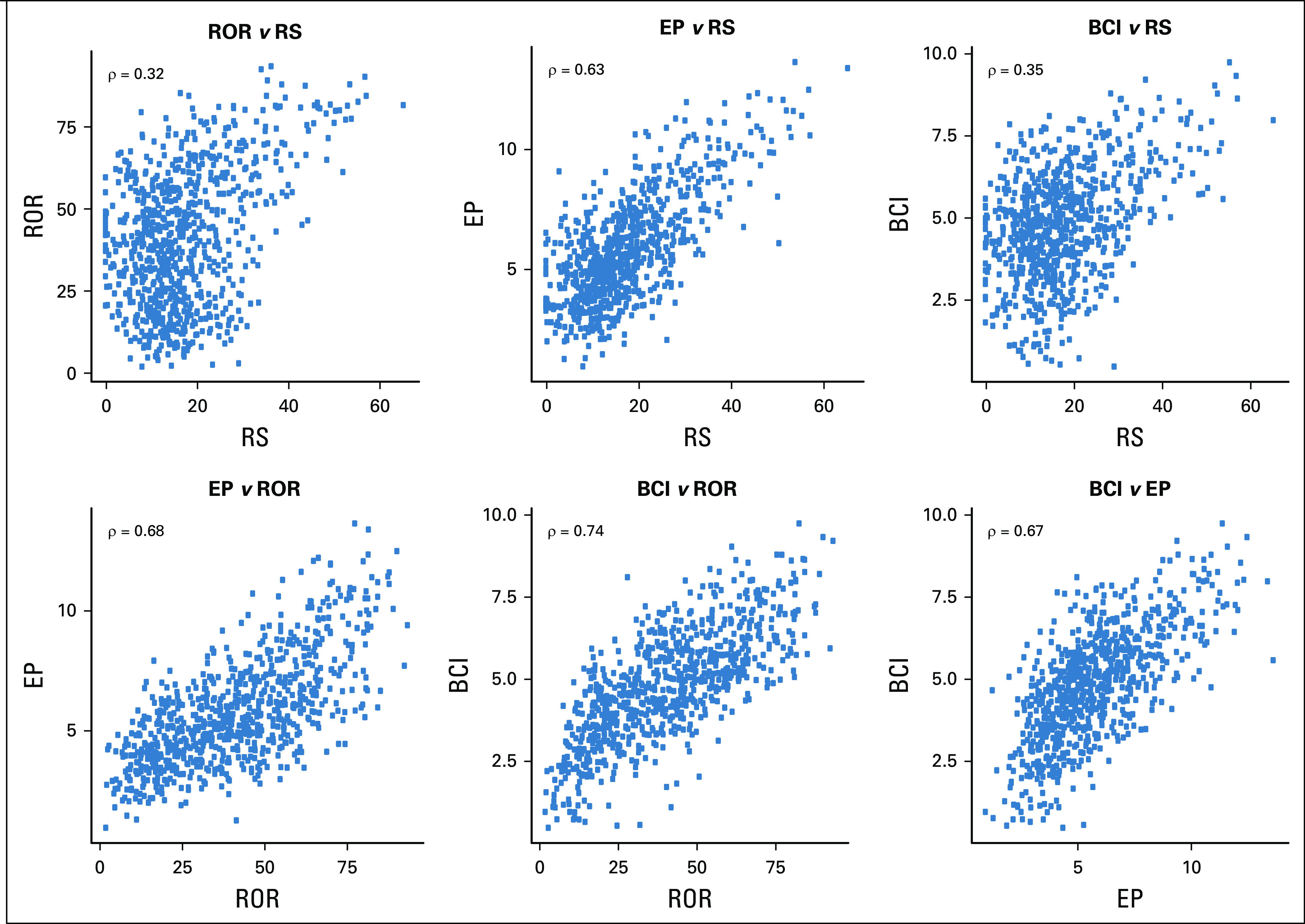
Scatter plots and Spearman’s ρ correlation coefficients of the Recurrence Score (RS), Risk of Recurrence (ROR), EndoPredict (EP), and Breast Cancer Index (BCI) molecular scores in TransATAC.

The correlation of the overall risk scores with one another is shown in Figure 2. RS correlated strongly with EP (ρ = 0.63) and moderately with ROR (ρ = 0.32) and BCI (ρ = 0.35) across the whole population. Nonetheless, almost all patients with an RS ≥ 31 had ROR and BCI scores above their respective median values (40.2 for ROR and 4.8 for BCI). Each of the other three scores correlated strongly with one another (ROR *v* EP, ρ = 0.68; ROR *v* BCI, ρ = 0.74; EP *v* BCI, ρ = 0.67).

### Relationship Between RS and Its Constituent Modules

**FIG 3.**
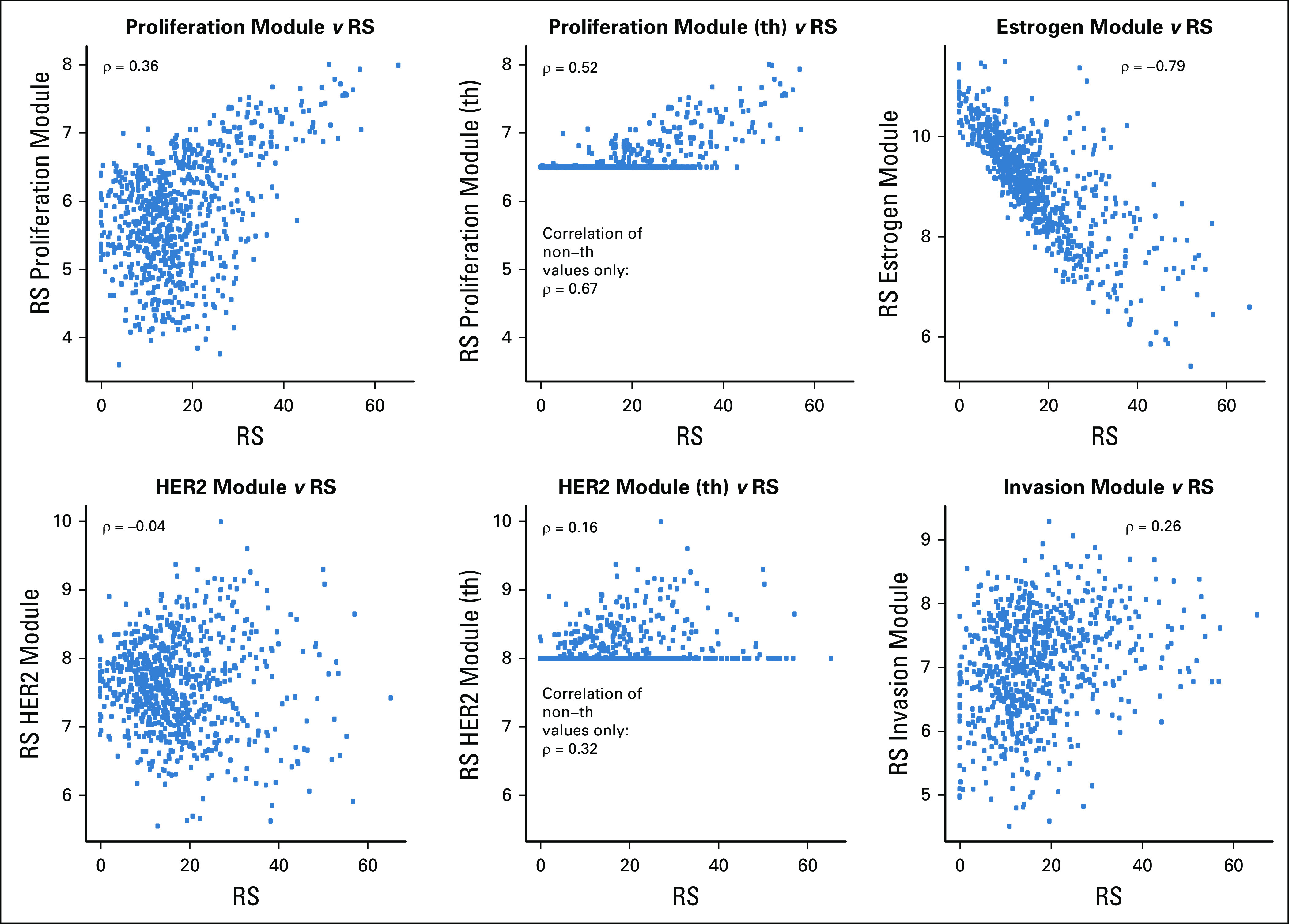
Relationship of the Recurrence Score (RS) with its module components. Spearman’s ρ correlation coefficients are presented. HER2, human epidermal growth factor receptor 2; th, thresholded.

To explore the molecular features driving the risk scores that may account for the similarities and differences between them, the RS modules were used in additional comparisons. The estrogen module had a strong negative correlation with RS (ρ = −0.79). There was a moderate correlation between RS and its proliferation module (ρ = 0.36; Fig 3). When the proliferation module had thresholding applied at 6.5 as in the RS algorithm (ie, any proliferation module value < 6.5 was adjusted to 6.5 by the algorithm), 614 (78.2%) of 785 samples were allotted a value of 6.5. The correlation between RS and the proliferation module with thresholding was ρ = 0.52; in the set of 171 samples where the threshold was not applied (proliferation module > 6.5), the correlation was ρ = 0.67.

**FIG A1.**
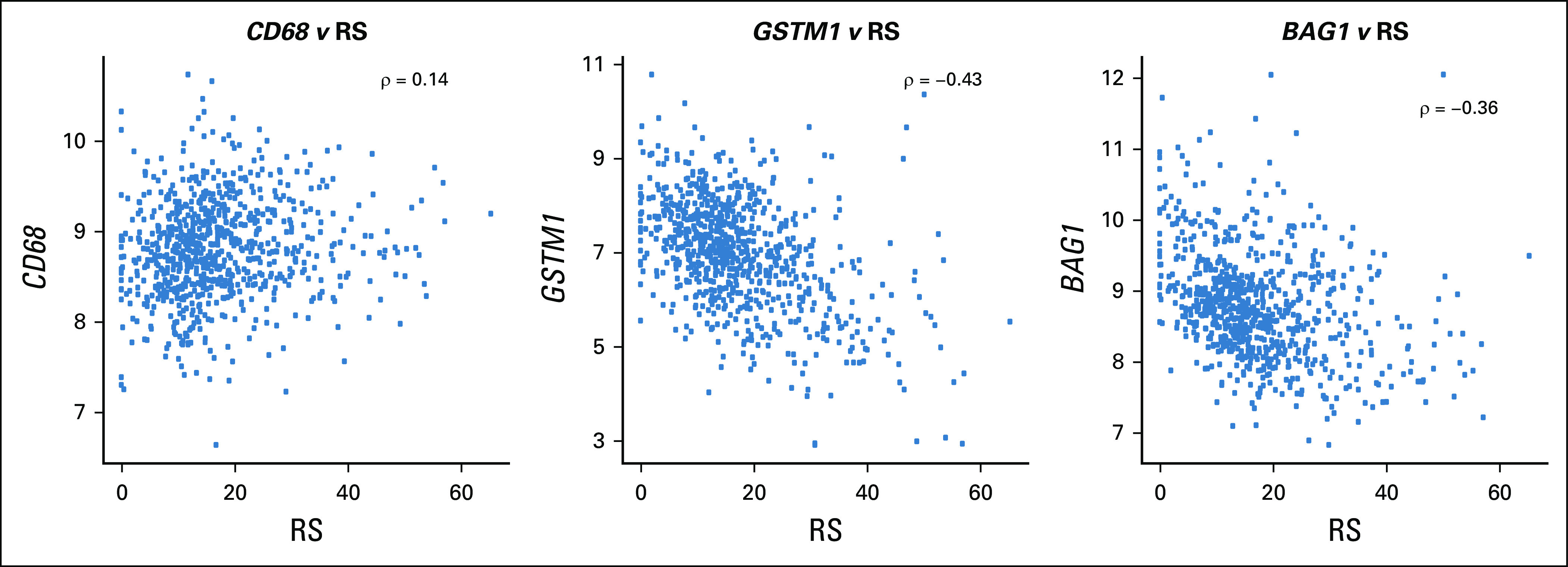
Associations of the Recurrence Score (RS) with the three RS genes not assigned to RS module: *CD68*, *GSTM1*, and *BAG1*. Spearman’s ρ correlation coefficients are presented.

The invasion module had a weak positive association with RS (ρ = 0.26). There was either no association or a moderate association between RS and each of the three individual genes not assigned to any of the modules (ρ = 0.14 with *CD68*; ρ = −0.43 with *GSTM1*; ρ = −0.36 with *BAG1*; Appendix Fig A1, online only).

The HER2 module had no association with RS (ρ = −0.04) in this HER2-negative cohort. Of the 785 samples, 573 (73.0%) had HER2 module scores < 8.0 and had the threshold value of 8.0 applied. There was no association between the HER2 module and RS when the threshold was applied (ρ = 0.16), and in the set of 212 samples above the HER2 threshold, the correlation with RS was ρ = 0.32.

RS’s comparisons with its components is not between independent measurements, and therefore, the observed correlations may be overstated. To perform a fair assessment of the contribution of the various molecular features to their overall score, we assessed how much of RS’s variance was explained by its module components. The estrogen module explained more than half of RS’s variance (59.1%), while the proliferation module accounted for approximately a fifth of RS’s information (19.4%; Table 2). In this cohort, both the invasion and the HER2 modules explained very little of RS’s variance (1.3% and 2.2%, respectively).

**TABLE 2.**
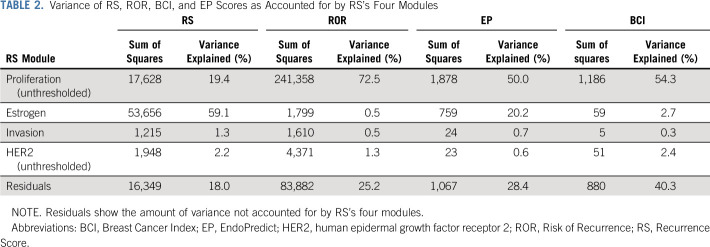
Variance of RS, ROR, BCI, and EP Scores as Accounted for by RS’s Four Modules

### Associations of ROR, EP, and BCI With the RS Proliferation, Estrogen, HER2, and Invasion Modules

**FIG 4.**
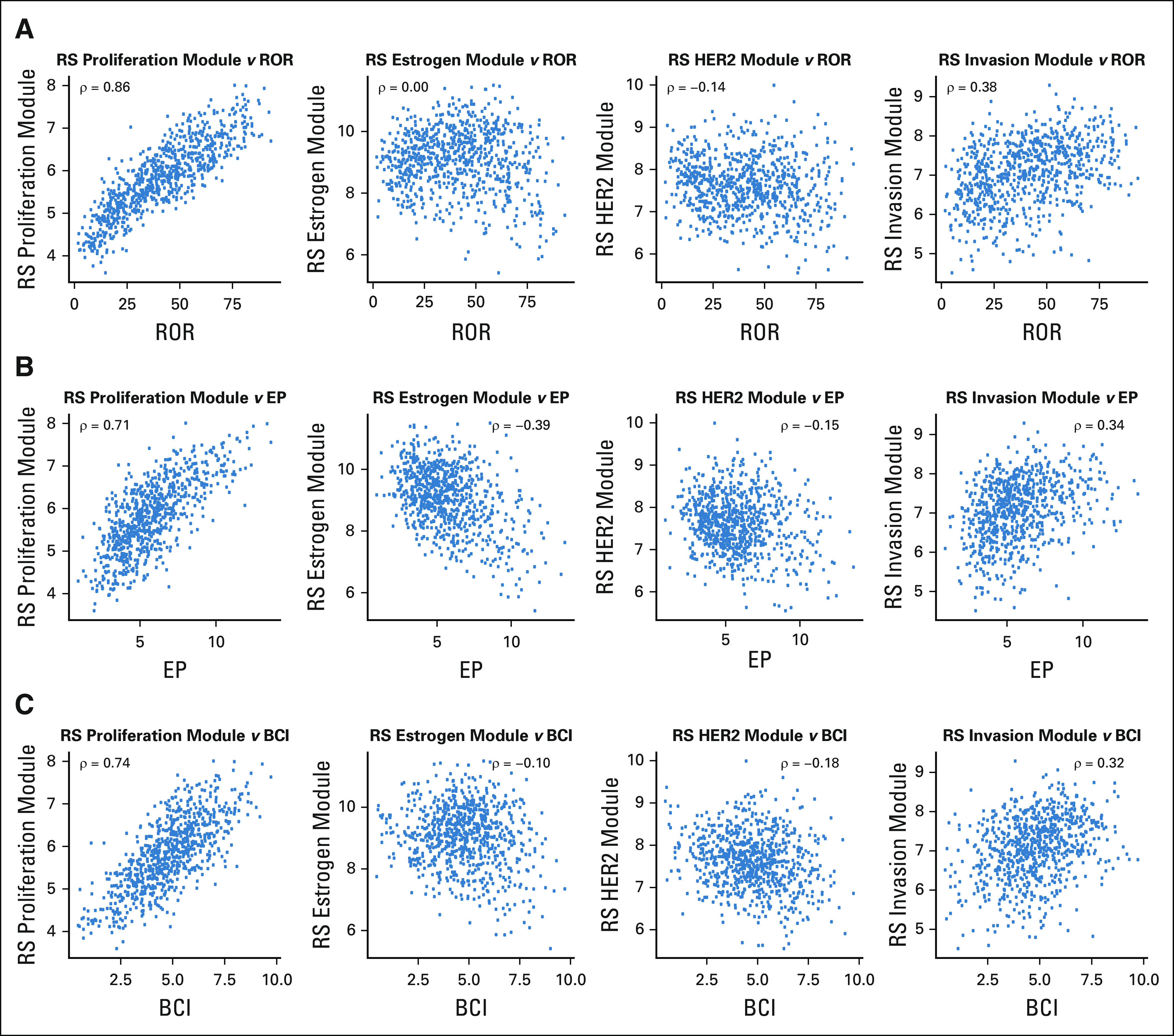
Associations of the (A) Risk of Recurrence (ROR), (B) EndoPredict (EP), and (C) Breast Cancer Index (BCI) with the proliferation, estrogen, human epidermal growth factor receptor 2 (HER2), and invasion modules of the Recurrence Score (RS). Spearman’s ρ correlation coefficients are presented.

We also analyzed the association of proliferation (unthresholded), estrogen, HER2 (unthresholded), and invasion-related RS module scores with each of the ROR, EP, and BCI scores. Most of the ROR score’s variance could be accounted for by RS’s proliferation module score (72.5%) and none by RS’s estrogen module score (0.6%; Table 2; Fig 4). Half of EP’s variance was accounted for by the proliferation module (50.0%) and an additional 20.2% by the estrogen module. The BCI score’s variation was largely accounted for by the proliferation module (54.3%) and almost none by the estrogen module (2.7%). Each of ROR, EP, and BCI had very little association with RS’s HER2 module (explained variance range, 0.6%-2.4%), and although the correlation of each of these with the invasion module was > 0.3, the explained variance ranged between 0.3% and 0.7%.

## DISCUSSION

The majority of patients with early ER-positive breast cancer will not experience a recurrence when treated with 5 years of adjuvant endocrine therapy.^16^ Finding the means to identify those patients such that they can safely be excluded from additional adjuvant chemotherapy and/or extended adjuvant endocrine therapy has been a high priority over recent years^17^ and has led to the development and widespread clinical use of several commercial multiparameter gene expression assays, including the four molecular assays studied here. It is therefore important that the oncologist interpreting and applying the scores for patient management have an understanding of the biologic features the tests reflect, particularly when trying to understand the difference among their readouts.

To our knowledge, this is the first four-way comparison of the assays in their commercial form aimed at evaluating their similarity and molecular drivers. Previously, we published the prognostic comparison of the four assays in their commercial form.^8^ A number of articles have compared some of the assays,^7,18-20^ but these have been of limited sample size and/or based around research assays for which comparability with the clinically used commercial assays is generally poorly documented. Discordant risk stratification in these studies, however, support our observations of varying agreement among the molecular scores.

The availability of the individual gene and gene module scores that constitute the Onco*type* DX RS provided a unique opportunity to study the degree to which the RS is driven by these individual features. Using the RS genes and gene modules as surrogate measures of the designated biologic features, we were also able to identify the major differences in the molecular drivers that lead to overall differences and similarities among the scores.

Of note, this study focused entirely on the molecular components of the tests and did not include the clinicopathologic features that are integrated into the final prognostic result for the ROR, EP, and BCI. We found that other than the relationship of each score with grade, they had little relationship with the other features we considered (age, tumor size, nodal status). Given that these features are known to have strong prognostic significance, their statistical independence from each of the scores underlines the potential for including them in a composite score for optimal prognostic estimates as we have previously published for the RS-pathology-clinical assessment.^21^ The stronger correlation of ROR, EP, and BCI (ρ = 0.45-0.50) than RS (ρ = 0.27) with grade probably reflects the weaker association of RS with proliferation.

The much weaker association of RS with its proliferation module than with its estrogen module is superficially surprising given that the coefficient in the Cox model is three times greater for the proliferation than for the estrogen module.^9^ This is at least partly explained by the score from the proliferation module being thresholded such that in this series, only 22% of the patients had the proliferation module unthresholded and therefore contributed meaningfully to the final score. Thresholding of the proliferation module presumably reflects the prognostic importance of the modules as measured in the training set of RS.^9^ Our earlier analysis of the effect of thresholding on prognostic estimates with the RS showed the proliferation module being more informative when thresholding was applied.^22^ Practitioners should therefore not be surprised if a high RS tumor is associated with low proliferation as reflected, for example, by immunohistochemistry for protein encoded by the *MKI67* gene or low grade as reported here.

One might conclude that this TransATAC population is of relatively good prognosis. Patients who had received chemotherapy (on the basis of clinicopathologic factors) were excluded such that outcome for endocrine therapy alone could be assessed. However, we found that it is similar to a SEER cohort of > 40,000 patients in terms of risk as determined by RS reported by Petkov et al.^23^ For patients with node-negative disease, 8.0% of the SEER population had RS ≥ 31, which was actually lower than the 10.4% for the TransATAC cohort. Similarly, among patients with node-positive disease, 7.0% of the SEER population had RS ≥ 31 compared with 11.1% in TransATAC (Appendix Table A3, online only). This is likely due to the lower chemotherapy use (9%) among UK patients on which the TransATAC collection is based compared with chemotherapy use in the overall ATAC cohort (21%).^24^ As a consequence, the cohort presented here includes many patients who would have received chemotherapy had they been treated in other countries.

The strong inverse correlation of the RS with the estrogen module indicates that its eventual score is likely to be particularly influenced by the benefit patients receive from their endocrine therapy. It may also explain the finding in our earlier reports in TransATAC where the RS showed a marked reduction in its prognostic performance between 5 and 10 years, while the ROR, BCI, and EP all showed continued substantial separation.^8^ In TransATAC after 5 years, the estrogen module had no prognostic significance,^22^ consistent with patients who showed the strongest estrogen signaling at diagnosis who lost much of their benefit from endocrine therapy at its withdrawal. Along with the pronounced importance of the estrogen module to the overall RS, this change at 5 years would be expected to lead to profoundly reduced prognostic performance of the RS after 5 years. The degree to which this observation in TransATAC may be generalizable is not known, but it is supported by the data related to the switching of the importance of estrogen signaling at 5 years being very similar to that reported by Bianchini et al.^25^ A meta-analysis from Early Breast Cancer Trialist Collaborative Group data of 74,194 women with primary ER-positive breast cancer who were scheduled to have received 5 years of endocrine therapy also found that higher progesterone receptor expression reflected better outcomes only in the first 5 years after surgery and not beyond 5 years.^26^

The strongest relationship of the other three tests was with the proliferation module of RS, with proliferation accounting for most of the variance between 50.0% for EP to 72.5% for ROR. The latter very high value may seem surprising given that the derivation of the ROR is related to the degree of correlation of a given tumor to each of the intrinsic subtypes.^27^ However, as well as these correlations, the algorithm includes a component that is determined by the expression of 18 proliferation-related genes in the 50-gene panel.^28^ Clearly, the weighting of that component results in scores in which proliferation is the dominant feature.

Our study has a number of strengths. The assays were carried out by the developers of the commercial assays who used their proprietary methods and were blinded to the patients’ clinical and outcome data. The same batch of RNA extracts were used for all four assays, which allowed for direct head-to-head comparisons of the scores and features. Weaknesses include that the module scores analyzed in this study are surrogates of the biologic features we assessed and that their direct role can be considered only with the RS. Nonetheless, the very strong relationships with proliferation and the individual genes of the estrogen module being those that are strongly related to estrogen signaling support the value of these surrogates. Furthermore, > 99% of the cohort was recorded as White, and this poses some limitation on the generalizability of the findings to racially more diverse populations.

In summary, we found that despite common thinking, the Onco*type* DX RS was primarily driven by estrogen-related rather than proliferation-related features in the majority of tumors in the TransATAC cohort. This is in contrast with the ROR, EP, and BCI that were dominated by proliferative features. The generalizability of these findings is supported by the similarity between the range of RSs in the analyzed population and that in the SEER database. These findings may explain the differences and similarities in the prognostic performance of these tests for early and late recurrence that we and others have reported.

## Figures and Tables

**TABLE A1.**
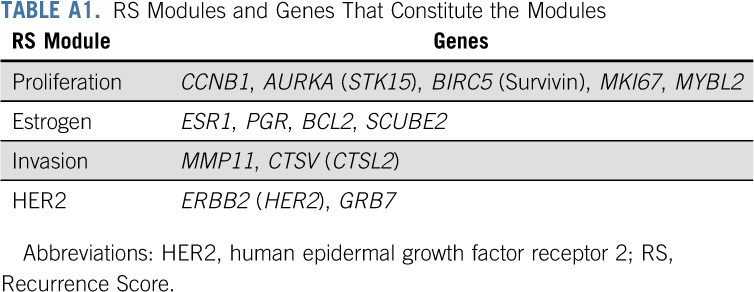
RS Modules and Genes That Constitute the Modules

**TABLE A2.**
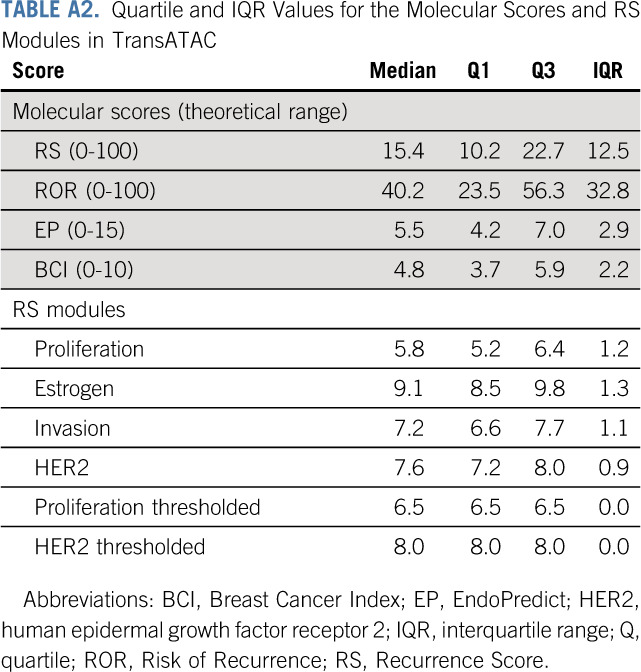
Quartile and IQR Values for the Molecular Scores and RS Modules in TransATAC

**TABLE A3.**
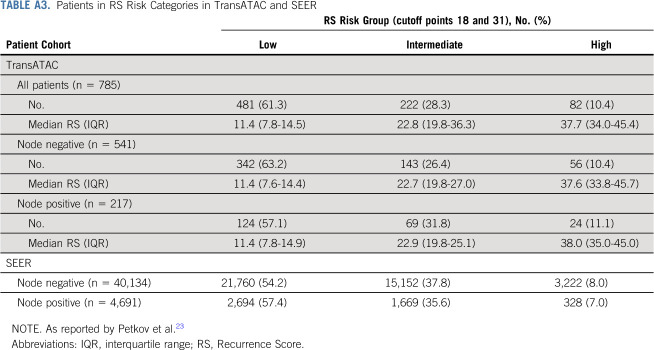
Patients in RS Risk Categories in TransATAC and SEER
